# A flexible data-driven audiological patient stratification method for deriving auditory profiles

**DOI:** 10.3389/fneur.2022.959582

**Published:** 2022-09-15

**Authors:** Samira Saak, David Huelsmeier, Birger Kollmeier, Mareike Buhl

**Affiliations:** ^1^Medical Physics, Carl von Ossietzky Universität Oldenburg, Oldenburg, Germany; ^2^Cluster of Excellence Hearing4all, Carl von Ossietky Universität Oldenburg, Oldenburg, Germany; ^3^Hörzentrum Oldenburg gGmbH, Oldenburg, Germany; ^4^Hearing Speech and Audio Technology, Fraunhofer Institute of Digital Media Technology (IDMT), Oldenburg, Germany

**Keywords:** auditory profiles, precision audiology, data mining, machine learning, patient stratification, audiology

## Abstract

For characterizing the complexity of hearing deficits, it is important to consider different aspects of auditory functioning in addition to the audiogram. For this purpose, extensive test batteries have been developed aiming to cover all relevant aspects as defined by experts or model assumptions. However, as the assessment time of physicians is limited, such test batteries are often not used in clinical practice. Instead, fewer measures are used, which vary across clinics. This study aimed at proposing a flexible data-driven approach for characterizing distinct patient groups (patient stratification into auditory profiles) based on one prototypical database (*N* = 595) containing audiogram data, loudness scaling, speech tests, and anamnesis questions. To further maintain the applicability of the auditory profiles in clinical routine, we built random forest classification models based on a reduced set of audiological measures which are often available in clinics. Different parameterizations regarding binarization strategy, cross-validation procedure, and evaluation metric were compared to determine the optimum classification model. Our data-driven approach, involving model-based clustering, resulted in a set of 13 patient groups, which serve as auditory profiles. The 13 auditory profiles separate patients within certain ranges across audiological measures and are audiologically plausible. Both a normal hearing profile and profiles with varying extents of hearing impairments are defined. Further, a random forest classification model with a combination of a one-vs.-all and one-vs.-one binarization strategy, 10-fold cross-validation, and the kappa evaluation metric was determined as the optimal model. With the selected model, patients can be classified into 12 of the 13 auditory profiles with adequate precision (*mean across profiles* = 0.9) and sensitivity (*mean across profiles* = 0.84). The proposed approach, consequently, allows generating of audiologically plausible and interpretable, data-driven clinical auditory profiles, providing an efficient way of characterizing hearing deficits, while maintaining clinical applicability. The method should by design be applicable to all audiological data sets from clinics or research, and in addition be flexible to summarize information across databases by means of profiles, as well as to expand the approach toward aided measurements, fitting parameters, and further information from databases.

## Introduction

It has become increasingly evident that characterizing hearing deficits by the audiogram alone is not enough. In addition to a loss of sensitivity, other factors, such as suprathreshold distortions, determine how well individuals can understand speech in daily life and communicate efficiently (1–5). However, it is yet an open issue which measures should be applied to achieve “precision audiology,” i.e., to characterize the individual patient as completely and exactly as necessary without losing too much time on comparatively irrelevant measurements. Hence, a number of approaches were described in the literature that differ in their general purpose, their amount of measurements included, and their evaluation method to characterize the most relevant measures.

For instance, van Esch et al. (6) proposed a test battery (“*auditory profile*”) for standardized audiological testing comprising eight domains (pure-tone audiometry, loudness perception, spectral and temporal resolution, speech perception in quiet and in noise, spatial hearing, cognitive abilities, listening effort, and self-reported disability and handicap) aiming to describe all major aspects of hearing impairment without introducing redundancy among measures. Similarly, the BEAR test battery was proposed for research purposes to characterize different dimensions of hearing and was evaluated with patients with symmetric sensorineural hearing loss (7). In spite of the benefit of the proposed test batteries, widespread adoption in clinical practice is currently lacking. The complete BEAR test battery, for instance, takes ~2.5 h to complete (7), even though a shorter version for clinical purposes was also proposed in (8). Nevertheless, in clinical practice, time is short and the assessment of patients on such a multitude of tests may not be feasible.

To tackle time constraints, Gieseler et al. (9) aimed at determining clinically relevant predictors for unaided speech recognition from a large test battery, thus, reducing the amount of required tests. They showed that pure-tone audiometry, age, verbal intelligence, self-report measures of hearing loss (e.g., familial hearing loss), loudness scaling at 4 kHz, and an overall physical health score were most important in predicting unaided speech recognition, with the pure-tone audiometry serving as the best predictor. Their model, however, left 38% of the variance in predicting unaided speech recognition unexplained, indicating that further measures may be related to unaided speech recognition. At the same time, their analyses were tailored toward explaining unaided speech recognition performance as an outcome measure. Predictors for aided speech recognition performance, in contrast, or other outcome measures, may vary. In Lopez-Poveda et al. (10), for instance, temporal processing deficits as measured by the frequency-modulation detection threshold (FMDT) were shown to be most relevant in predicting aided speech recognition performance. When including only predictors available in clinical situations, however, the unaided speech recognition threshold (SRT) in quiet was determined to be the best predictor. This demonstrates the discrepancy between research and clinical applications and highlights the importance to analyze insights from both clinical and research datasets in combination. It further shows that the relevance of predictors depends on the outcome measures, as different predictors were determined most relevant for unaided and aided speech recognition.

To improve patient characterization in the field of audiology, patient data, therefore, need to be summarized efficiently and flexibly. By summarizing patient data flexibly, the generated knowledge could be used in a variety of settings (e.g., in clinics, for mobile assessments, and decision-support systems in general), and for a variety of outcome measures (e.g., diagnostic outcomes or unaided and aided speech recognition performance). This, however, poses several challenges. First, patients need to be characterized across different dimensions of hearing loss. Second, to gain insights from a diverse patient population, data aggregation across databases is required, which, however, is hindered by the heterogeneity in the applied measures across clinical and research databases in the field of audiology (11). Lastly, for the general applicability of the stored information, it needs to be accessible *via* measures also applied in clinical settings, such that physicians can be supported.

To tackle these challenges, different approaches toward patient stratification exist that involve identifying subgroups in patient populations based on measurement data from single measures or from interrelations of measures. An example of a data-driven stratification based on single measures is the Bisgaard standard audiograms by (12). There, a set of 10 standard audiogram patterns occurring in clinical practice were defined. This has subsequently resulted in a variety of studies investigating outcome measures such as aided SRTs in relation to the 10 audiograms [(9, 13–15), to name a few], aiming toward precision audiology, thus, demonstrating the promising nature of finding sub-classes in the field of audiology. In contrast, an expert-based approach, based on single measures, was proposed by Dubno et al. (16) that linked four audiometric phenotypes to knowledge about possible etiologies from animal models of presbyacusis *via* expert decisions. Schematic boundaries for the five phenotypes “older-normal,” “pre-metabolic,” “metabolic,” “sensory,” and “metabolic+sensory” are provided which allow for inferences of etiologies, given patient presentations of presbyacusis.

In contrast to patient stratification based on single measures, Sanchez-Lopez et al. (17) introduced a data-driven profiling method based on multiple measures using a combination of unsupervised and supervised machine learning. Based on the hypothesis that two distortion types for the characterization of hearing loss exist, four distinct profiles were generated by means of principal component analysis and archetypal analysis. Thereby, the most important variables for the characterization of each distortion dimension were estimated and employed to identify the most extreme data combinations (archetypes). All patients of two existing research data sets (containing a certain battery of tests) were labeled with the most similar archetype. In a second step, decision trees were built to allow for the classification of new patients into the four auditory profiles. The obtained profiles are interpretable as they were defined based on the hypothesis of two distortion components and the variables used for classification are known. The meaning of the two distortions, however, was different depending on the available measures in the respective data set.

Sanchez-Lopez et al. (18) improved the profiling method to be more robust (e.g., due to bootstrapping, a more flexible number of allowed variables, and estimating the association of a patient to a profile based on probability) and applied it to the BEAR test battery (7), which was designed for the purpose of including all relevant measures according to the literature and previous work. As a result, a plausible interpretation of the two distortion dimensions was obtained, namely being associated with speech intelligibility and loudness perception, respectively (18). However, by tailoring their analyses toward four extreme distinct profiles and by using archetypal analysis, a priori hypotheses were included in the derivation of the profiles. Consequently, further distinctions between patient groups may be lost.

A further example of summarizing audiological data efficiently is provided by Buhl et al. (11, 19). The Common Audiological Functional Parameters (CAFPAs) were derived by experts and aim at representing audiological functions in an abstract and measurement-independent way. The CAFPAs further act as an interpretable intermediate layer in a clinical decision-support system. Prediction models allow for a data-driven prediction of CAFPAs (20) and a subsequent classification into audiological findings (21). However, to relate new measures from further data sets to the CAFPAs, experts are currently required for labeling purposes, which consequently does not allow for the automatic integration of new data sets containing additional measures.

The aforementioned methods all contribute toward enhancing patient characterization but are either restricted to single measures or include prior assumptions regarding the distinction of patient groups or audiological functions. Consequently, not all existent differences between patient groups may be detected. In this study, we aim at (1) providing a method for a fully data-driven stratification of patients into subgroups based on audiological measures, namely *auditory profiles*. This patient stratification approach is not restricted in terms of prior assumptions, the number of patient groups, and contained measures. In that way, all differences between patient groups can be summarized independently of outcome measures. The auditory profiles aim to describe patient groups with similar measurement ranges across audiological measures and are defined based on the contained patient patterns, instead of prior assumptions. In future, profiles could, hence, be combined, added, or removed, depending on the provided insights gained from applying the profiling approach to further data sets, as well as based on the relevance of profile distinctions in clinical routine. The applicability of defined profiles to different settings (e.g., clinical settings) can, however, only be obtained if the knowledge from within the profiles, in the form of plausible ranges for the contained measures, can be linked to patients, given their results on widely used measures (e.g., pure-tone and speech audiometry). We, therefore, further aim at (2) maintaining clinical applicability by building classification models using random forests, based on measures available in clinical routine. This allows for classifying new patients into the auditory profiles. In clinics, it could support physicians to associate a new patient to a profile and in that way exploit statistical knowledge available for the respective profile.

The current study, thus, aims at answering the following two research questions:

RQ1: Does our proposed profiling approach result in a meaningful and distinct grouping (auditory profiles) of patients with respect to important hearing loss factors contained in the employed data set?

RQ2: Which classification model can provide high precision and sensitivity in classifying patients into the auditory profiles using only a subset of the contained audiological measures?

## Materials and methods

### Data set

To define the first set of auditory profiles, we analyzed an existing data set that was provided by Hörzentrum Oldenburg gGmbH and is described in detail in Gieseler et al. (9). In contrast to Gieseler et al. (9), we did not exclude any patients with, e.g., an air-bone gap >10 dB HL but aimed for a diverse patient sample. Our patient sample, consequently, consisted of all patients that completed the full test battery, resulting in 595 patients (mean age = 67.6, SD = 11.9, female = 44%) with normal to impaired hearing. For each patient, information with respect to a broad range of measures, including audiogram data, loudness scaling, speech tests, cognitive measures, and anamnesis questions is contained.

The contained measures either are, or can easily be integrated into clinical routine. The audiogram and the Göttingen sentence test (GOESA) (22) are commonly used for the assessment of individuals' hearing status. The former assesses an individual's thresholds across frequencies; the latter assesses the speech recognition threshold (SRT), here, in noise for the collocated condition (S0N0). Both the audiogram and the GOESA are used in hearing aid fitting, for gain adjustments, and as an outcome measure, respectively. From the contained measures, we used several features to generate the auditory profiles (see Table 1 for an overview of the features). For the audiogram, the pure-tone average (PTA, threshold averaged across 0.5, 1, 2, and 4 kHz) for air-, and bone conduction was used for the more severely affected ear. Asymmetric hearing loss was accounted for *via* the inclusion of an asymmetry score (absolute difference between PTA of left and right ear). Additionally, the air–bone gap (ABG), the PTA of the Uncomfortable Loudness Level (UCL), and the Bisgaard standard audiograms (12) were derived from the audiogram. The Bisgaard standard audiograms were included to allow for a separation of different audiogram patterns (e.g., moderately and steeply sloping audiograms), while reducing the dimensionality of the audiogram. A further speech test [digit triplet test (DTT)] (23) was included to add information to the auditory profiles from a measure mainly used for screening purposes. The adaptive categorical loudness scaling (ACALOS) (24) provides relevant information with respect to an individual's loudness perception and recruitment, and has also shown its effectiveness in hearing aid fitting (25). To characterize both the lower and upper part of the loudness curves, both L15, L35, and the difference between L15 and L35 were selected as features. As a relation between cognition and hearing exists (26), the age-normed sum score from a screening test for dementia (Demtect) (27) and the raw score from a measure of verbal intelligence [Vocabulary test (WST)] (28) were also included. Further, information regarding the socio-economic status (sum score of education, income, and occupation) (29), the presence of tinnitus [none (1), unilateral (2), bilateral (3)], and the age of the patients were available.

**Table 1:** Overview of audiological domains and features used for the generation of the profiles.

**Domain**	**Number of features**	**Features**
Audiogram	6	**AC PTA**, BC PTA, **Asymmetry (left/right ear)**, ABG, UCL PTA, **Bisgaard standard audiograms**
Loudness Scaling	6	**ACALOS (L15,L35, L15-L35) for 1.5 & 4 kHz**
Speech tests	3	**GOESA (SRT, slope)**, DTT (SRT)
Cognitive measures	2	DemTect score, WST score
Anamnesis	3	Tinnitus, Socio-economic status, **age**

Features used for the classification into the profiles are shown in **bold**.

### Generating auditory profiles using model-based clustering

**Figure 1:**
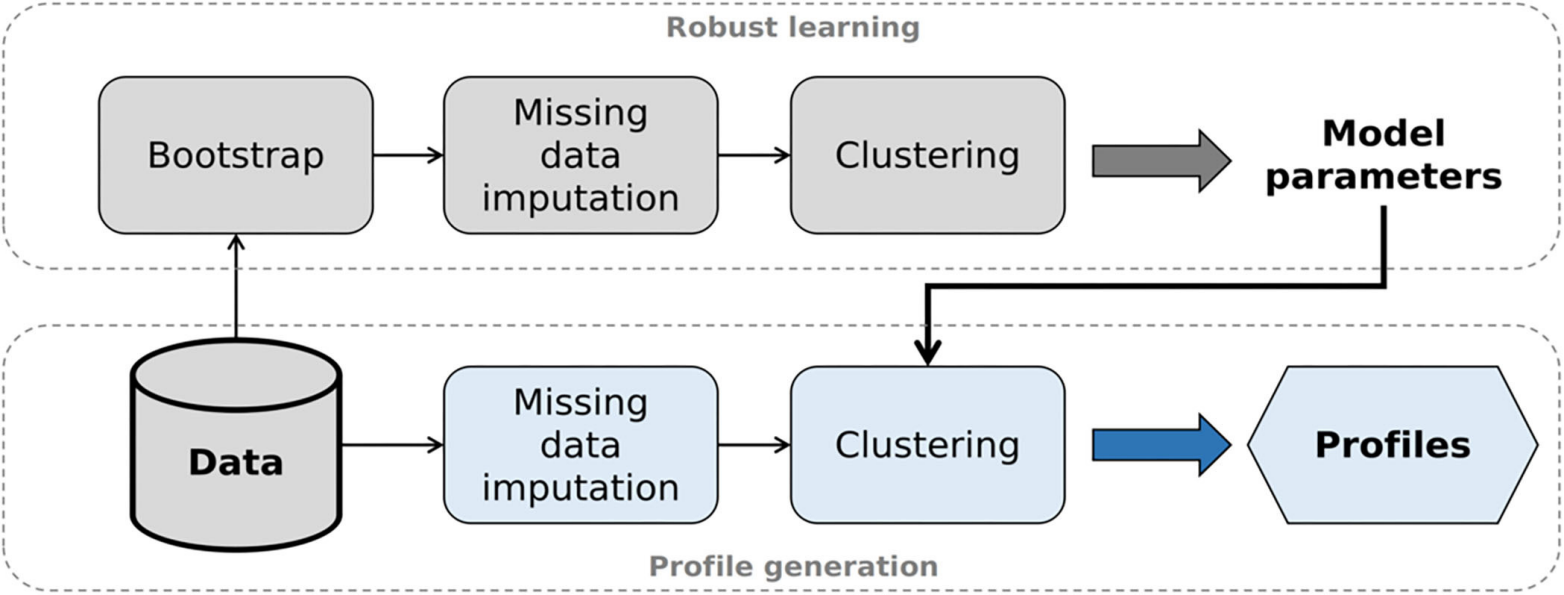
Analysis pipeline to generate auditory profiles. After selecting the optimal model parameters (robust learning, upper part), model-based clustering is applied to the original data set (profile generation, lower part).

To generate auditory profiles that are capable of separating patients with respect to ranges of audiological tests, we applied clustering, as it has shown promising for purposes of patient stratification. For the current analyses, the clustering pipeline consists of two steps, namely robust learning and profile generation (see Figure 1 for visualization).

#### Robust learning

##### Bootstrapping and imputation of missing data

As bootstrapping techniques have shown to improve the robustness of clustering solutions (30, 31), we first subsampled the data set 1,000 times containing 95% of the original data set. We chose subsampling over resampling with replacement, in order to avoid duplicate samples being seen as a “mini”-cluster, hence, artificially increasing the number of clusters. As missing values existed in the original data set, each of the 1,000 subsamples also contained missing values and needed to be imputed. Missing values pose a common problem in clinical data sets, and a loss of patient information, e.g., complete-case analysis, is often undesirable, thus, requiring an adequate technique to solve it.

Consequently, for audiogram data, prior to extracting pure-tone averages and Bisgaard standard audiograms, missing thresholds were interpolated if the thresholds prior to and after missing values were available. For the remainder of missings (on average 1.5% with a maximum of 2.5%), multivariate imputations with chained equations (MICE) (32) was applied. MICE results in multiple completed data sets that account for the uncertainty that stems from imputing missings. With MICE, the analyses of interest are subsequently performed on all completed data sets and the results are combined (32). For the present analyses, we generated 20 completed data sets. Accordingly, clustering was performed on each of the 1,000 × 20 data sets.

##### Model-based clustering

Before clustering, we transformed the features of Bisgaard standard audiograms and tinnitus and treated them as continuous for clustering purposes. Bisgaard standard audiograms were ordered with respect to increasing PTA; tinnitus with respect to its absence, unilateral, or bilateral presence. All features (see Table 1) were then scaled using min–max scaling, resulting in values between 0 and 1. As the number of features (*N* = 20) can be considered small, we refrained from further dimensionality reduction and instead aimed at maintaining a balance of the number of features stemming from the different measures. Depending on the clustering goal, dimension reduction with, e.g., principal component analysis can prove problematic as the reduction of dimensionality could also lead to the removal of information that would have proved to be discriminatory for the clustering goal (33).

On the scaled feature set, we applied model-based clustering. Model-based clustering was especially suitable for our purposes of uncovering patient groups existent in the data set, as it assumes that the data stem from a mixture of subgroups. The mixture of subgroups is further assumed to be generated by an underlying model which model-based clustering aims to recover (34, 35). For this purpose, the number of clusters *k* and a parameterization of the covariance matrices with respect to their shape, size, and orientation [see (36) for possible covariance parameterizations] need to be specified beforehand. Subsequently, each cluster's mean vector *mu*_*k*_ and covariance matrix Σ*k* is learned and a likelihood estimate for the given clustering solution is computed.

In contrast to simpler clustering techniques such as k-means clustering, model-based clustering is able to detect more complex shapes in the data (37). It is, therefore, more suitable for our purposes of detecting all plausible differences in the data. At the same time, the parameterization of the covariance matrices can constrain the complexity of the clustering solution by enforcing stronger restrictions and reducing the number of parameters that need to be estimated (38). To select the most suitable model, all candidate parameterizations (*k* and covariance matrix parameterization) are computed and the model with the highest likelihood of explaining the underlying data structure is selected using the Bayesian Information Criterion (BIC) (39). More complex clustering structures (i.e., less covariance matrix restrictions) may suffice in explaining the dataset with fewer clusters but require the estimation of a much larger number of parameters and are, thus, not always feasible with smaller datasets. Less complex clustering structures, in contrast, could explain the same underlying data structure by increasing the number of clusters (38). This also holds for increasing the number of features used for clustering. Increasing the number of features increases the number of parameters to be estimated (i.e., the complexity), which, however, can be reduced by restraining the covariance matrices. This may, in turn, increase the number of estimated clusters required to explain the data. To avoid increasing the number of clusters beyond clusters that enhance the explanation of the data structure, however, the BIC penalizes for the complexity of the covariance parameterization and number of clusters *k*, and thus, results in a trade-off between model complexity and over-parameterization (34).

Here, for each of the 1,000 × 20 data sets, we computed all potential parameterizations for 2–30 clusters and then derived the optimal model for each data set using the BIC, which resulted in 1,000 × 20 candidate models. The dimensionality of the candidate models was then reduced across the 20 completed data sets of each of the 1,000 subsamples. The most frequently occurring model parameterization was selected as a candidate model, resulting in a reduced set of 1,000 candidate models. We then defined the overall optimal model *via* its frequency across the 1,000 candidate models, which resulted in an estimate for the model parameters (i.e., the number of profiles and the model's covariance parameterization).

#### Profile generation

In the profile generation step, we generated the auditory profiles using the original data set without prior subsampling. First, we imputed missings using multivariate imputation with chained equations (MICE) in the same manner as described in Section Bootstrapping and imputation of missing data. Thus, 20 completed data sets were generated with differing estimates for missings. Second, we applied model-based clustering using the estimated optimal model structure from the robust learning step for each completed data set, which resulted in 20 candidate clustering solutions. From these 20 candidate clustering solutions, we aimed to select the solution showing the highest overlap with the remaining solutions regarding patient allocation into the clusters. The rationale behind this is that, since model parameters are kept constant, differences between clustering solutions stem from differences in the imputed values. The solution showing the highest overlap can then be assumed to be least influenced by imputed values, as patient allocations into the clusters were agreed upon by most solutions.

### Building classification models to classify patients into auditory profiles

#### Features and labels

To allow for the usage of the auditory profiles for different purposes (e.g., clinical applications), it is necessary to classify patients into the profiles based on a subset of measures widely available. Therefore, we built classification models using the profiles as labels and a reduced set of measures as features. From the aforementioned features used for clustering (see Table 1), only the features from ACALOS, GOESA, and the air-conduction audiogram (PTA, Asym PTA, Bisgaard) were used next to the age of the patients (12 features), to simulate the case that these measures were conducted for a to-be-classified patient.

#### Model training

For model training, we split the reduced data set, containing the above-mentioned 12 features, into a training (75% of patients) and test data set (25% of patients). The training data set was used for training the model, which included cross-validation (CV), model tuning, and the selection of the best model tuning parameters containing different binarization strategies, CV procedures, and evaluation metrics defining the prediction error, and are described in more detail in the following. The best model is defined as the model minimizing prediction error. We then evaluated the training data set's best model on the test data set to estimate its predictive performance on patient cases not used for model training, which indicates how the classification model would generalize on unseen patient cases.

To build the classification models on the training data set, we used random forests (40), as it has shown competitive classification performance, while remaining interpretable. It is also less prone to overfitting and handles relatively small sample sizes well (41, 42). Random forests are an extension of simple decision trees. Multiple decision trees are built, each segmenting the predictor space into several smaller regions, based on derived decision rules. Predictions are consequently derived from the ensemble of trees. For classification purposes, the label predicted most frequently among trees is selected. In other words, it has the highest estimated probability among candidate labels. To avoid building correlated trees, the tuning parameter *mtry* defines the number of features considered at each split. At each split, the specified number of features is then randomly sampled from the feature set, thus, enforcing different tree structures, which in turn reduce the variance of the predictions (41). For the current analyses, we tuned *mtry* using cross-validation.

To provide optimal prediction models for each of the profiles, we applied different binarization techniques. Binarization strategies to tackle multi-class problems have proved beneficial in enhancing predictive performance. They involve building base learners for binary classification tasks which are subsequently aggregated to provide a prediction (43, 44).

Consequently, we compared multi-class classification to three different binarization strategies. First, we built predictive models for each auditory profile separately (*k* models), with the one-vs.-all (OVA) technique, allowing the model to learn the specific differences of a profile, as compared to all remaining ones. Thus, for each profile, we built a classification model that decides whether a patient belongs to a given profile, or not. If more than one of the *k* OVA models predicted that a patient belonged to its profile, the profile with the highest probability among candidate profiles is selected, as defined by the frequency of its prediction in the random forest. Second, we used a one-vs.-one (OVO) technique to build predictive models for all *k*(*k*-1)/2 profile combinations. Thus, differences between each pair of profiles were learned. To provide a prediction, voting aggregation was applied, which means that the most frequently predicted profile was selected. Lastly, we used a combination of OVA and OVO (OVAOVO). Here, again, we used OVA to predict profile classes. However, for uncertain cases, if more than one profile was predicted, instead of selecting the profile with the higher probability, we used OVO to decide upon the final profile prediction.

Across profiles, a class imbalance exists, either due to differing profile sizes or due to the applied binarization strategy. Classifiers trained on imbalanced data sets tend to favor the majority class over the minority class in order to reduce the prediction error, which leads to undesirable results if the minority class is of interest (e.g., in an OVA or OVO model). Consequently, we upsampled all profiles to contain at least the number of patients of the largest profile *p* in terms of sample size (*maxN*_*p*_). Upsampled patients were selected randomly from each profile and across features Gaussian noise was added to the observations (+/- 1 *SD*). Upsampling with Gaussian noise was shown to be especially suitable for clinical data sets (45). As a result, no class imbalance was present for multi-class and OVO. For OVA, the class imbalance was still present due to the OVA design. As upsampling would require upsampling for several magnitudes of the original profile size, and downsampling would discard too much valuable information, a different technique was applied. In addition to upsampling to *maxN*_*p*_,we used a weighted random forest model using cost-sensitive learning. Thus, weights were introduced, which more severely punished for the misclassification of the minority class over the majority class (46). The issue of the tendency toward majority predictions was, therefore, addressed also for the OVA binarization strategy.

Further, we compared two different CV schemes for optimal model tuning, namely, leave-one-out CV (LOOCV) and 10-fold CV repeated 10 times (RepCV). LOOCV is a special case of CV, in which the validation set consists of only one observation; RepCV splits the training set randomly into 10-folds, which is then repeated 10 times. LOOCV provides advantages for small data sets, as models are trained on larger sample size as compared to RepCV. However, in return, predictions may have high variance, as the variation in training sets is small. RepCV, in contrast, has lower variance due to differing training sets, but may be biased due to smaller sample size (41).

Lastly, we compared different evaluation metrics which optimize classifiers to different aspects of predictive performance. The main measures to evaluate the performance of a classifier are accuracy, sensitivity, specificity, and precision. Accuracy defines the ratio between correctly classified instances and the total sample size. Sensitivity (also called recall) and specificity are evaluation metrics for binary classification problems, but can be easily extended toward multi-class classification problems by employing an OVA binarization of the classification problem. This, however, again introduces an imbalance in the data regarding the evaluation. Sensitivity refers to correctly classifying all classes of interest as positive, whereas specificity refers to the ability to correctly classify all remaining classes as negative. The precision of a classifier, in contrast, determines the preciseness of a classifier. That means precision is high if no other class was misclassified as the class of interest (47). The four evaluation metrics we compared in the current study, namely, Cohen's kappa, balanced accuracy, F1-score, and the Area under the precision–recall curve (AUPRC) differently weight aspects of accuracy, sensitivity, specificity, and precision. Cohen's kappa is inherently capable of evaluating multi-class problems, by comparing the accuracy to the baseline accuracy obtained by chance (48). Balanced accuracy weights sensitivity with specificity, and is consequently less able to handle multi-class problems, since specificity increases with imbalanced data sets. The F1-score addresses this issue by calculating the harmonic mean between sensitivity and precision, instead of sensitivity and specificity. Likewise, the AUPRC has shown to be especially suitable for imbalanced data (49). To determine the optimal classifier, it is important to select an adequate evaluation metric, suitable for the class distribution in the data set. Since we have different class distributions across our four classification strategies (multi-class, OVA, OVO, OVAOVO), we compared different evaluation metrics.

#### Model selection and evaluation

To select the optimal classification model, we evaluated the four different classification strategies (multi-class, OVA, OVO, OVAOVO) on the training data set with respect to the different metrics (Kappa, balanced accuracy, F1-score, and AUPRC) and cross-validation procedures (repCV, LOOCV). To compare the performance of the models that were optimized with the different evaluation metrics, after training, a general *post-hoc* performance measure is needed. Here, we chose the F1-score as it summarizes both sensitivity and precision, and can adequately describe the performance of a classifier in case of imbalance. Accordingly, we determined the model leading to the highest F1-score by averaging the F1-scores across profiles and then selected it as the best performing classification model. Lastly, to evaluate the predictive performance of the selected classification model and its generalizability to new data, we evaluated the model on the test data set. Here, instead of the F1-score, we used both sensitivity and precision to provide a more thorough assessment of the classifiers' performance for the distinct auditory profiles.

## Results

### Generation of profiles

#### Estimation of profile number and covariance parameters

**Figure 2:**
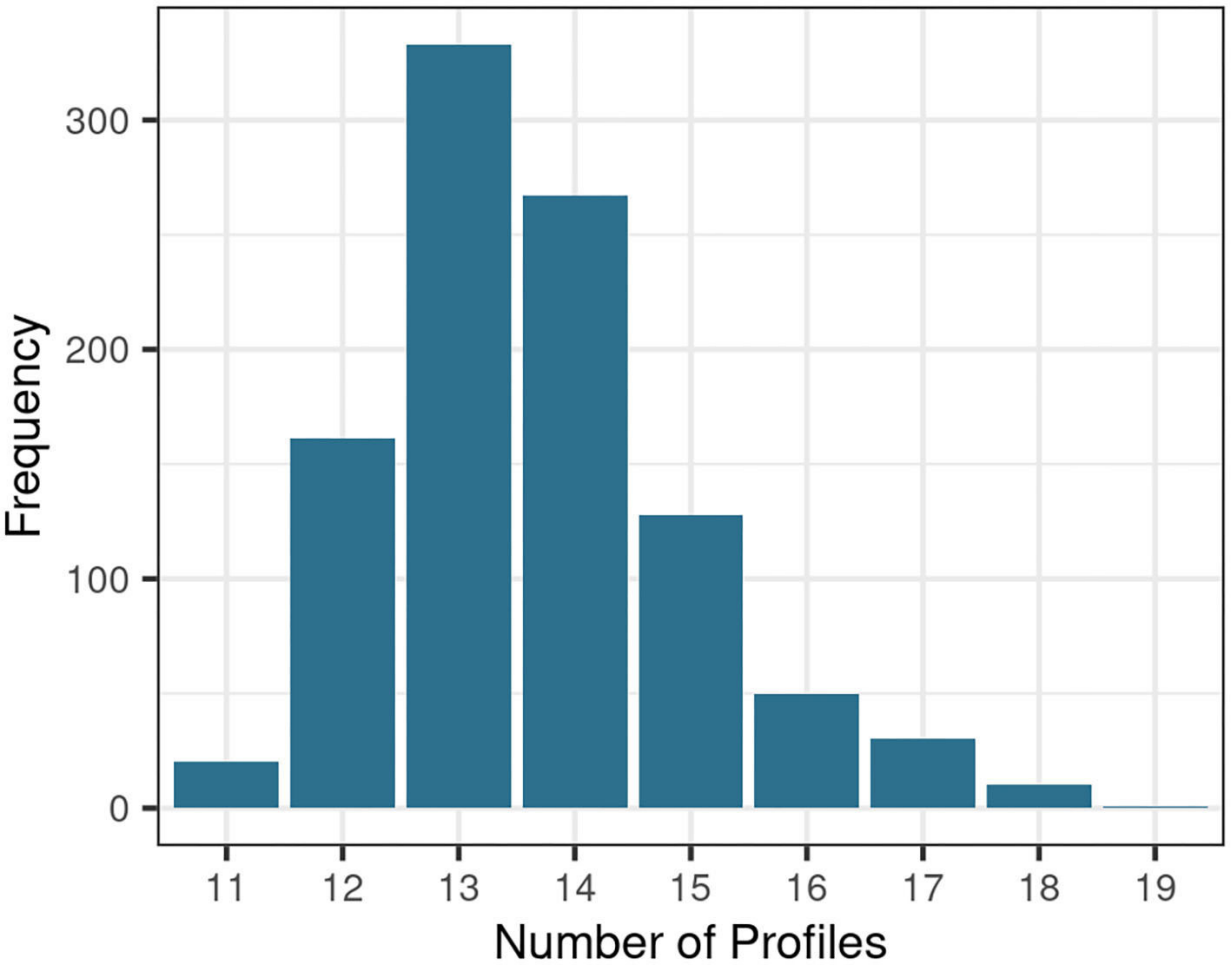
Distribution of optimal profile numbers across bootstrapped samples.

To generate auditory profiles which characterize a diverse range of patient patterns across measures, the number of separable patient groups and the covariance parameter were determined. Figure 2 depicts the distribution of estimated cluster numbers across the 1,000 bootstrapped samples. Across bootstrapped samples, 11–19 profiles were estimated as an optimal model with a maximum of 13 clusters. Further, the covariance parameterization “VEI” was selected across all 1,000 subsamples. VEI (variable volume, equal shape, coordinate axes orientation) is a rather parsimonious model as it restricts both the shape and axis alignment of the clusters and requires a diagonal cluster distribution. The sizes of the clusters, however, may vary. Hence, 13 clusters with the covariance parameter “VEI” are estimated to represent the data structure best.

Subsequently, the above-defined parameterization (*k* = 13, “VEI”) was used to generate profiles on all 20 completed data sets of the original data set. The completed data set showing the highest overlap with the remaining completed data sets regarding patient allocation into the profiles (*max_similarity* = 0.794) was selected to base the auditory profiles on. Mean classification similarity across all 20 completed data sets was 0.75 (*SD* = 0.032).

#### Profile ranges across audiological measures

**Figure 3:**
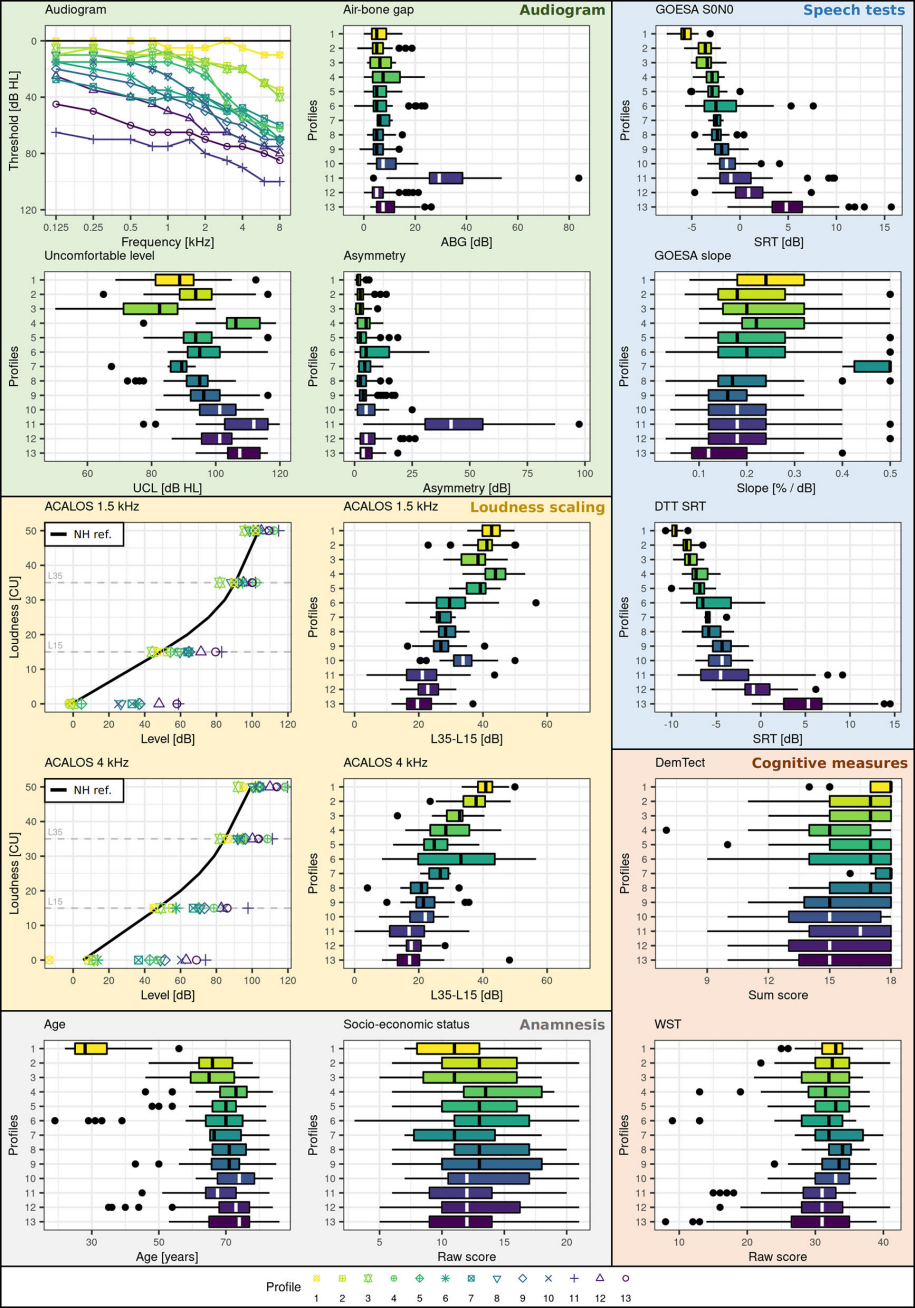
Profile ranges across measures. Plot backgrounds are colored according to underlying domains. Blue corresponds to the speech domain, green to the audiogram, yellow to the loudness domain, orange to the cognitive domain, and gray to the anamnesis. Profiles are color-coded (yellow to violet) and numbered (1-13) with respect to increasing SRT (impairment) on the GOESA.

Figure 3 shows the profile ranges of the generated auditory profiles and Table 2 contains the number of patients contained in each profile. The profiles cover a large range across audiological measures and show profile-based differences in patient presentation of the contained measures. All profiles can be distinguished from each other based on at least one audiological feature. The speech test results (Figure 3, blue box) regarding GOESA and the DTT are generally comparable. The profiles cover different extents of impairments, ranging from normal hearing (profile 1) to strong difficulties in understanding speech in noise (profile 13), as indicated by the increasing SRT. Likewise, the slope of the GOESA decreases with increasing SRT. Within the SRT range of −5 to 0 dB SNR, most of the profiles are contained. Here, the different profiles show similarities regarding SRT ranges, and the difference between the profiles can be found *via* other measures. Audiogram results (Figure 3, green box) indicate the existence of normal hearing (profile 1), moderately (profiles 2, 3, 6, 7, 8, 9, 11, 13), and rather steeply sloping (profiles 4, 5, 10, 12) patterns. Generally, we observe a trend of increasing thresholds on the audiogram together with increasing SRTs. There are, however, also exceptions. Profile 11 displays the highest thresholds across frequencies and profiles, but does not show the strongest impairment on the GOESA. Instead, it includes patients with an air–bone gap and asymmetric hearing loss, as indicated by the asymmetry score. Profiles can also be distinguished based on the ACALOS (Figure 3, loudness scaling—yellow box) and the UCL. With increasing SRTs, we can observe an increase in the UCL, as well as a decrease in the dynamic range, as shown by the difference between L35 and L15 for both 1.5 and 4 kHz. In spite of this, differences exist across profiles unrelated to the increasing SRT. Profiles 4 and 5, for instance, show overlapping ranges regarding the SRT, but differ with respect to the UCL. Across cognitive measures (Figure 3, cognitive measures—orange box), no clear distinctions across profiles were found. Likewise, ranges for the age of patients and the socio-economic status (Figure 3, anamnesis—gray box) overlap across profiles, with the exception of profile 1 containing younger patients.

**Table 2:** Number of patients contained in each auditory profile.

**Profile**	**1**	**2**	**3**	**4**	**5**	**6**	**7**	**8**	**9**	**10**	**11**	**12**	**13**
*N*	27	76	19	24	77	33	6	44	68	51	42	79	39

To summarize, similarities exist to varying extents between profiles. Some profiles can be easily distinguished. For instance, profiles 1 and 2 can be easily distinguished from profiles 11, 12, and 13 across audiogram, GOESA, and loudness scaling data. In contrast, other profiles only differ on certain measures. Profiles 2 and 3, for instance, show overlapping ranges on both the audiogram and the GOESA, but different average loudness curves and distinct distributions regarding the UCL.

### Classification into profiles

#### Model selection

To allow for a classification of new patients into the auditory profiles based on a reduced set of measures widely available in clinical practice, classification models were built using random forests. Different parameterizations (optimization metrics, binarization strategies, and CV procedures) were compared with the aim to provide the classification model best suited for the auditory profiles. The *mtry* parameter was inherently determined within each model.

**Figure 4:**
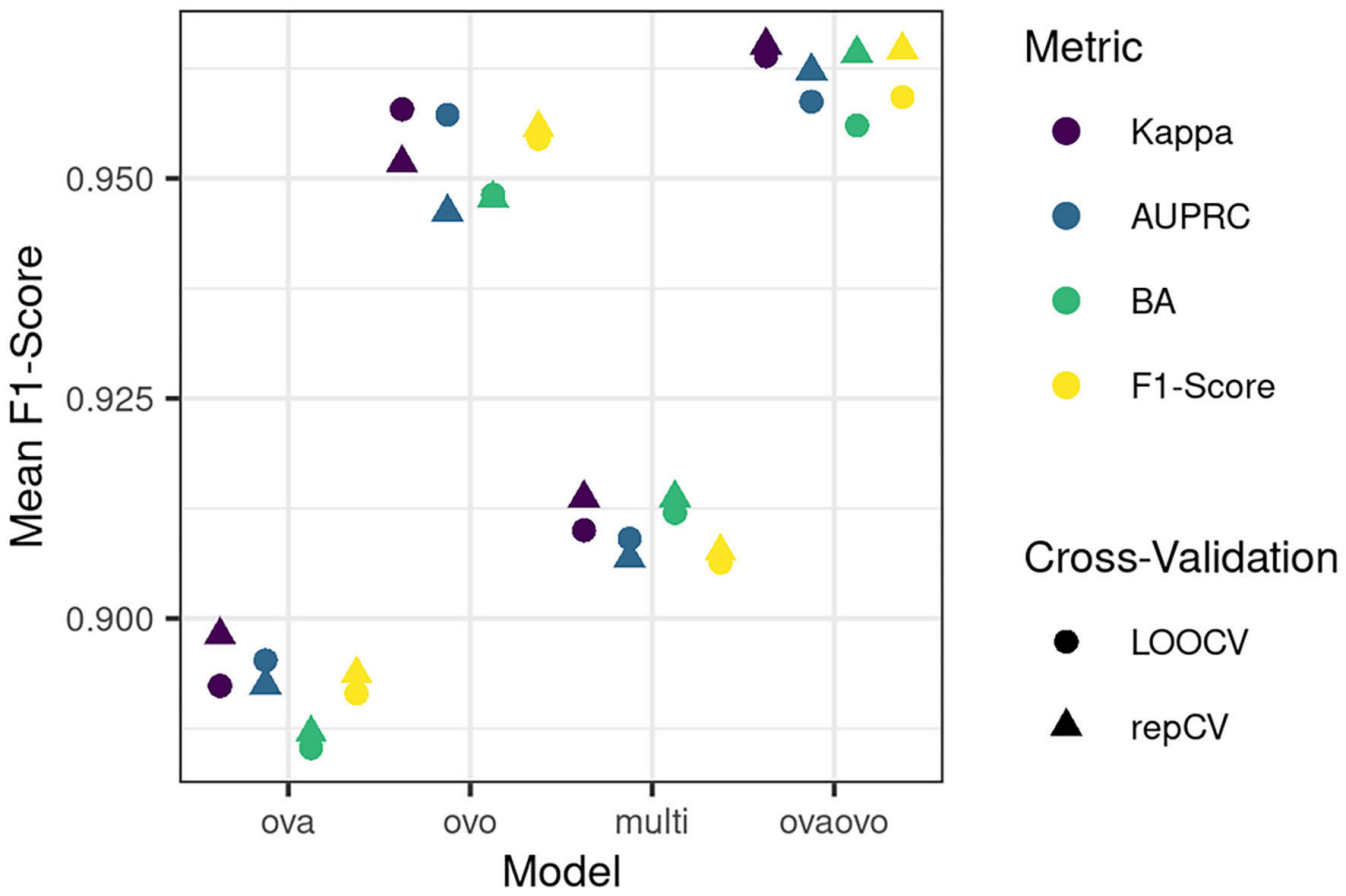
Performance of different models on the training data set. The mean F1-score was calculated as the mean of F1-scores across profiles 1–6 and 8–13. Metrics and cross-validation schemes can be distinguished by color and shape, respectively. BA refers to balanced accuracy. LOOCV refers to leave-one-out cross-validation; repCV to repeated 10-fold cross-validation.

Figure 4 displays the results of the comparative performance with respect to the binarization strategies, optimization metric, and cross-validation procedure on the training data set. Model performances with respect to the F1-scores were averaged across profiles to result in an overall F1-score. This allowed for a selection of the best model parameterization. Profile 7 was not selected for averaging, as the number of patients contained in the profile (*N* = 6) is not large enough to lead to reliable results and interpretations.

All models perform well in predicting profile classes, as indicated by the overall small and high range of mean F1-scores. The highest F1-score was obtained by the OVAOVO model using the kappa evaluation metric and repeated 10-fold CV. Consequently, the OVAOVO (kappa, repCV) model is selected as the classification model to allow for a prediction of patients into profiles. Across models, the kappa metric provided the best results, whereas optimal CV procedures differed across binarization strategies, with the exception of the OVAOVO model in which repCV provided the best results for all evaluation metrics.

#### Model evaluation

**Figure 5:**
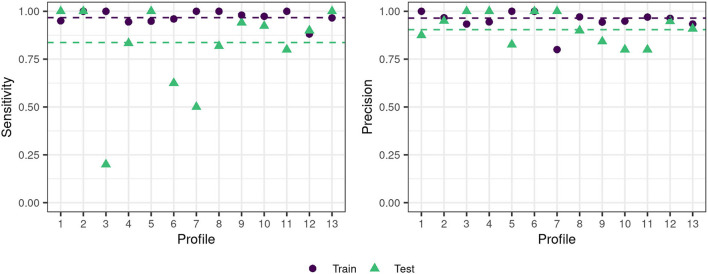
Train-test data set performance for the OVAOVO (kappa, repCV model) for both sensitivity and precision. The dashed lines indicate the mean across profiles 1–6 and 8–13 for the respective condition.

The previously selected optimal model (OVAOVO, repCV) was selected based on its performance on the training data set (75% of the patients). To investigate the generalizability of the classification model to new patients, its performance was subsequently evaluated on the test data set (25% of the patients). Figure 5 displays the performance results with respect to the sensitivity and precision across all profiles.

Generally, the classifier's performance is adequate regarding achieved sensitivity and precision on the test data set. Across profiles 1–6 and 8–13, average precision and sensitivity on the test data set are 0.9 and 0.84, respectively. Results for profile 7 were plotted for completeness, however, are unreliable due to the small sample size, since the test data set only consisted of two patients. Overall test performance is only slightly lower than training performance for most profiles, except for profiles 3, 6, and 7. For these profiles, the generalization of the learned classification approach toward unseen data is limited. Profile 3 and profile 6 show low levels of sensitivity, but high levels of precision. Thus, not all cases of the two profiles are detected, however, if the two profiles are predicted one can be highly certain that the patient does, indeed, belong to profile 3 or profile 6.

## Discussion

The aim of this study was to propose a flexible and data-driven approach to patient stratification in the field of audiology that allows for a detailed investigation into the combination of hearing deficits across audiological measures. Our results demonstrate the feasibility and efficiency of our proposed profiling pipeline in characterizing hearing deficits in the form of patient groups, namely, auditory profiles. The proposed 13 auditory profiles separate patients with respect to ranges on audiological tests. Further, to ensure the applicability of the auditory profiles in clinical practice with only a basic set of audiological tests, classification models were built that allow for an adequate classification of the auditory profiles given such a reduced set of audiological measures.

### Generation of profiles

The proposed profiles aim to represent the underlying patterns of the current data set best. Hence, the profiles describe the patterns across measures for the available patients and etiologies, rather than aiming to cover all generally existent patient groups with the current set of auditory profiles. Additionally, the number of profiles that can be generated is variable and dependent on the underlying data. This becomes evident when inspecting the distribution of optimal profile numbers in Figure 2. Across bootstrapped data sets different profile numbers were suggested. This may in part be due to the applied method. Different subsets of the bootstrapped data may miss extreme patient patterns, and thus, lead to a reduction or increase in suggested profile numbers. This, next to the added uncertainty that stems from imputing missings, may explain the variability in suggested profile numbers across bootstrapped samples. By using a bootstrapping approach, where the optimal number of profiles is defined as the most frequently proposed profile number, it can be assumed, however, that the effects of imputations and extreme patient patterns on the generated profile number were minimized.

The number of profiles may further be influenced by the employed model restrictions. Since the covariance parameterization “VEI” restricts both the shape and axis alignment and requires diagonal cluster distributions, a parsimonious model was selected as describing the underlying data structure best. The number of profiles, therefore, may be large in order to characterize the data structure best with the given restrictions (38). It would be of interest to apply the modeling approach to a larger dataset that allows for a less restrictive model in order to investigate if the resultant number of profiles would decrease. A more parsimonious model that leads to a larger number of profiles, however, is in line with our aim of detecting all plausible differences between patient groups.

### Interpretation of profiles

The profiles, generally, cover a large range of different types and extents of hearing deficits and appear audiologically plausible. All profiles can be distinguished from each other by at least one audiological feature and can, thus, be considered as distinct patient groups regarding audiological measures (RQ1). The relevance of the distinction has to be evaluated with respect to the outcome measure of interest. Certain distinctions are, for instance, not necessarily relevant for diagnostic purposes. It can be assumed that profiles 4 and 5 would be categorized as bilateral sensorineural hearing loss (ICD code h90.3) (50) and could, thus, for purposes of coarse diagnostic classification be combined. Profile 5, however, shows a lower range of UCL levels, indicating that loudness would need to be compensated differently in a hearing aid for patients within profile 5 as compared to profile 4. The distinctions regarding loudness perception could influence the benefit that patients within the separate profiles may experience from hearing aids, if the same hearing aid parameters are applied to both groups. This highlights our motivation for flexible profiles that can be combined or separately considered given different outcome measures. The exact number of profiles may, therefore, change with the inclusion of further datasets and also depend on the targeted outcome measure. The proposed auditory profiles, however, enable a detailed investigation into differences that exist between patient groups.

Most of the profiles can be assumed to be caused by symmetrical sensorineural hearing loss. Profile 11, however, also contains an asymmetric conductive hearing loss, as indicated by the presence of both an asymmetry between the ears and an air–bone gap in the group (51). For the remainder of the profiles, however, we can interpret the profiles in the consideration of the four-factor model for sensorineural hearing loss by Kollmeier (52). The current profiles contain measures that allow for an estimation of the first two factors (attenuation and compression loss), but not binaural and central loss. The audiogram can provide an indirect indication for the attenuation loss, which is defined as the required amplification for each frequency to obtain an intermediate loudness perception (L25), whereas the ACALOS can indicate a compression loss *via* a reduced dynamic range (52). Overall, we can observe differences in both the audiogram shapes and the dynamic ranges across profiles. Most importantly, similar audiogram shapes (e.g., profiles 2 and 3) do not necessarily lead to a similar compression loss and our profiles are able to detect these differences, which is in line with the assumption of the four-factor model, that the audiogram alone cannot explain all underlying characteristics of sensorineural hearing loss. We, therefore, conclude that the 13 auditory profiles provide meaningful information regarding two important factors of hearing deficits, i.e., attenuation and compression loss (RQ1), and that the profiling pipeline has the potential for the detection of patient group differences also for further datasets, if suitable measures are included.

In general, the interrelation across speech tests, loudness scaling, and audiogram data lead to a separation of patients into profiles. For instance, profiles 2 and 3 contain patients with both similar SRTs and audiogram thresholds. Profile 3, however, shows a reduced dynamic range with its uncomfortable loudness level (UCL) thresholds derived from the audiogram and the range between soft (L15) and loud (L35) sounds on the ACALOS reduced, which indicates recruitment. This, in turn, has implications for hearing aid fitting. It can be assumed that patients within the two profiles require different compression settings, in spite of similar audiograms (53, 54). In contrast, the main difference for profiles 8 and 9 lies within their thresholds on the audiogram, with profile 9 showing about 10 dB higher thresholds, while showing similar SRT and loudness curve ranges. The relevance of a distinction between these two profiles, for both diagnostics and hearing aid fitting, thus, needs to be further investigated. For other profiles, differences are more strongly pronounced and they can well be separated.

Certain profiles also align well with the proposed phenotypes by Dubno et al. (16). Profiles 6, 7, and 9 are consistent with the metabolic phenotype, and profiles 2 and 3 appear to be in between the pre-metabolic and metabolic phenotype with respect to the ranges on the audiogram. Profile 4 can be described in terms of the sensory phenotype and profiles 5 and 10 as the metabolic + sensory phenotype. However, the auditory profiles also contain different patterns, with either more severe presentations as described by the phenotypes (profiles 11 and 13), or different slopes in the lower frequency range of the audiogram (profiles 8 and 12). Further, instead of an older normal hearing profile to match the older normal hearing phenotype, only a young normal hearing profile is included. Regardless, certain probable etiologies can be inferred for the respective profiles, exemplifying how alternate stratification approaches could be connected to the auditory profiles proposed in this study. Since more than one profile can be matched to sensory and metabolic phenotypes, however, it can, again, be assumed that further contributors regarding individual presentations of hearing deficits exist, which are not assessed *via* the pure-tone audiogram.

No distinctions across profiles regarding the cognitive measures were found (WST, DemTect). Even though hearing deficits and cognitive impairments have been widely associated (55), the precise causal relationship remains unclear and some studies did not find significant relations (26). With the profiles, a slight trend toward increasing impairment on the DemTect with increasing SRT can be observed; however, the ranges across profiles overlap substantially. On the one hand, this may indicate, that none of the present profiles is significantly influenced by cognitive abilities and that the observed patterns of hearing deficits may occur for both cognitively impaired and non-impaired patients. This would require further investigations and the inclusion of patients with more severe cognitive impairments. On the other hand, the DemTect, as a screening instrument, may not be sensitive enough for detecting a further association between cognitive impairment and hearing deficits. For the auditory profiles, this indicates that cognitive differences are not well-represented, such that patients' cognitive abilities would need to be assessed *via* further cognitive measures that are currently not included in the database.

The currently available profiles naturally only provide a picture of the contained measures. It can be assumed that the inclusion of further measures will enhance the precision of patient characterization. Of the specified eight domains relevant for characterizing hearing deficits, defined by van Esch et al. (6), currently, four are contained in the defined profiles (pure-tone audiogram, loudness perception, speech perception in noise, and cognitive abilities). Spatial contributors, i.e., the intelligibility level difference (ILD) and binaural intelligibility level difference (BILD) measures, were—unfortunately—not included in the original database so no relation to the profiles given here can be provided. However, it can be assumed that they could provide an enhanced characterization of patients' hearing status, as well as prove valuable for hearing aid fitting. Similarly, measures describing the central factor of hearing loss could be incorporated if available in a data set, to comply with all four factors as suggested by Kollmeier (52). Consequently, future studies should work toward incorporating these measures into the profiles.

### Classification into profiles

By building classification models to match patients into the auditory profiles using only features from the air-conduction audiogram, loudness scaling, and GOESA, we aimed for the applicability of the profiles in a variety of settings. First, in clinical routine, both the audiogram and a speech test, measuring the SRT, are the current standard in hearing aid fitting (56), and in Germany, the GOESA is included in the German guideline for hearing aid fitting (57). In addition, loudness scaling has proved promising for hearing aid adjustments (58). The three measures are, therefore, often available for hearing professionals and do not extend the testing time of patients and physicians. If fewer measures are available, e.g., only the audiogram and the GOESA, or a different set of measures, the classification models would have to be retrained for this purpose. We believe, however, that loudness scaling provides valuable information for hearing aid fitting and should, thus, be included in the fitting process. Second, to use the profiles in further research and clinical data sets, it is important to include measures that are frequently measured and available. Thus, even though further measures may be contained in the data sets, it is necessary to provide classification models containing measures widely available across data sets.

The present results indicate the feasibility of classifying patients into most of the profiles. The OVAOVO model with the kappa loss function and 10-fold repeated CV reached the highest F1-score and was, therefore, selected as the optimal classification model for the analyzed dataset. With the model test set, sensitivity was >75% for all profiles but profiles 3, 6, and 7 (RQ2). For profile 7, this can be explained by the small sample size of the profile as only six patients were classified into the profile. Consequently, the training of a classifier for profile 7 does not lead to reliable results, and its generalizability is not assured. In spite of that, we included the results for profile 7 for completeness, since it may provide further separation from the remaining profiles for the multi-class classifier, by including counter-examples of patients. Profile 7, however, cannot yet reliably be used to classify new patients into it. Further information from databases is needed to investigate whether this profile represents rare cases or whether this profile was not represented enough in the present data set to provide a large enough sample size for classification purposes. Profile ranges for profile 6 are generally broader than for other profiles; therefore, misclassifications may have occurred more frequently, thus, reducing the sensitivity for profile 6.

The current classification model naturally only covers patient populations that were also contained in the analyzed dataset. Given the adequate classification performance of the classifier, it can be assumed that new patients with similar characteristics to the patients within the dataset would also be adequately predicted into the auditory profiles. At the same time, random forests allow for an estimation of the classification uncertainty when classifying patients into the profiles. This uncertainty estimation refers to how often a patient was predicted into a given profile across the decision trees of the random forest as compared to the remainder of the profiles. For certain predictions, there is a high amount of agreement of the random forest, whereas for uncertain predictions there is a lower amount of agreement of the random forest. New patients are, therefore, classified into a given profile with an estimate of uncertainty, which, in turn, could also indicate if none of the profiles adequately represents the given patient. This could then reveal a rare patient case or a patient belonging to an additional profile that has not yet been defined. Generally, patients would always be allocated to a profile based on all measures that are contained in the classification model (i.e., audiogram, ACALOS, age, GOESA) and no single feature would determine the classification. For instance, the analyzed dataset contains mainly elderly hearing impaired patients and younger normal hearing patients. Children and younger individuals may, however, also experience hearing deficits. A classification based solely on the feature age would lead to a misclassification into the normal hearing profile 1. The generated classification model, in contrast, would also consider information from the audiogram, ACALOS, and GOESA and in that way avoid misclassification into the normal hearing profile 1.

It can be argued that predictive performance would have been improved by including all measures in the classification models. However, we aimed at providing classification models that can be readily used with measures available across clinics in Germany, such that no additional testing is required and time constraints of physicians are met. Consequently, we decided on a reduced set of measures and aimed at predicting profiles with widely available measures. In future, it may be of interest to provide classification models for all combinations of measures, such that if, e.g., bone-conduction thresholds or more specific psychoacoustic tests are also available in clinical settings, they can be used to increase predictive performance with regard to, e.g., the “binaural” and “central noise” factor (52) involved in characterizing the individual hearing problem.

One limitation of the present classification is the number of patients contained in each profile. For further validation larger and more balanced data sets that also contain more severe patients are required, which can also be assumed to lead to improvements in the predictive performance. An increase in the size of the training set will support the training of the classifier, whereas an increase in the test set will improve the certainty of the predictions. Currently, test performance may have been artificially high for some profiles due to the small sample size in the test set. However, further reducing the training size would also not be desirable, as it would increase the bias of the classification models. Thus, further evaluations on additional data sets containing further patients are required.

### Properties of the profiling approach and comparison to existing approaches

The current data-driven approach toward generating auditory profiles to characterize patient groups is not aimed at being contradictory with hitherto available profiling approaches but aims at providing a more detailed account of existing patient groups and offers several advantages.

First, its flexibility in the definition of profiles derived *via* purely data-driven clustering allows extending and refining the profiles, if in further data sets more extreme patient representations are contained. More specifically, it can be assumed that applying the profiling approach to additional data sets containing both similar and more extreme patient presentations will result in a set of auditory profiles that show overlap to herein proposed profiles, but also contain additional profiles. The new set of profiles could then be used to update the current set of auditory profiles. As a result, the total number of auditory profiles is not fixed and instead remains flexible to include further profiles. Likewise, the presented profiling pipeline can be applied to additional data sets with varying measures. In case of differing measures across data sets, measures not used for clustering purposes could serve as descriptive features and allow for inference, if these features occur more frequently in certain profiles. The flexibility in terms of derived profiles and contained measures could, in future, aid in comparing patients across data sets. Appropriate means to combine profiles generated on different data sets, however, need to be defined. For this purpose, a profile similarity index based on, e.g., overlapping densities (59) could provide a cut-off score on when to combine or extend profiles.

Second, profiles are not tailored toward a certain outcome such as diagnostics or hearing aid fitting. This may, in part, explain the rather large number of generated profiles, since profiles may differ with respect to measurement ranges but not with respect to audiological findings, diagnoses, or treatment recommendations. By tailoring our analyses toward certain outcomes, we could have possibly reduced the number of generated profiles. Our aim, however, was to generate as many profiles as plausibly contained within the data set such that all differences between patient groups can be caught. More specifically, by using Bisgaard standard audiograms also as a feature for clustering, patients were already separated into 10 distinct audiogram ranges. Combining 10 separate audiogram ranges with different loudness curves and SRT ranges already leads to a larger amount of profiles, if these patterns across measures and patients (i.e., profiles) occur frequently and are well-distinguishable from other profiles. At the same time, the flexibility of the profiles by their definition directly on measurement ranges allows reducing the number of profiles if only certain outcomes are of interest. For instance, if, in future, profiles are connected to diagnostic information from further data sets, profiles leading to a distinction with respect to a diagnosis could be separated or merged. Similarly, if profiles are used for hearing aid fitting, only those profiles leading to separable groups with respect to aided parameters could be retained.

Third, all patients can be grouped into auditory profiles. In contrast, in Dubno et al. (16), around 80% of audiogram shapes were categorized as non-exemplar and could not be matched into one of the phenotypes, whereas in Sanchez-Lopez et al. (18), an “uncategorizable” category in addition to the four profiles exists.

A fourth advantage of the flexibility of our auditory profiles pertains to its ability to provide complementary knowledge compared to other profiling approaches, which allows analyzing the same data sets from different perspectives and potentially learning more about the inherent patterns. To exemplify, the profiling approach by Sanchez-Lopez et al. (18) is applicable to different audiological data sets as well and also comprises the two steps of profile generation and classification. Both approaches are data-driven; however, the approach by (18) is based on the hypothesis of two distortion types which limits the number of profiles to four. In contrast, our approach is purely data-driven, that is, the obtained number of profiles directly depends on the available combinations of measurement ranges in the respective data set, in order to detect all existing differences between patients. Each of our profiles (estimated by model-based clustering) characterizes the group of included patients in terms of underlying measurement data, while the profiles of (18) are characterized by one respective extreme prototypical patient (due to archetypal analysis) and all other patients classified into a respective profile show less extreme results on the variables identified by principal component analysis. The profiles of (18) are interpretable due to the hypothesis of two distortion types and the variables related to each distortion type; however, the obtained interpretation depends on the available measures in the dataset. That means that it needs to be ensured to employ an appropriate database, as was achieved in Sanchez-Lopez et al. (18) with the BEAR test battery (7), following the findings of (17) where the choice of data led to different, not completely plausible interpretations based on the two different analyzed datasets. In contrast, our profiling approach does not include explicit interpretability of every profile yet, but instead, interpretability needs to be added as an additional step. This can be done by relating the profiles to the literature as discussed above, or by including expert knowledge to label the different profiles. In addition, the type of interpretability required for different outcome measures considered in future analyses may be different, and can then be chosen appropriately.

For associating the profiles obtained by the two approaches, in a first step, the distributions of patient data grouped to profiles can be manually compared, for instance regarding audiogram and SRT ranges in Figure 6 of (18) and in our Figure 3. However, this comparison is limited as only a small subset of measures is common in the BEAR test battery and our dataset, as well as due to methodological differences as discussed above. Instead, it would be interesting to apply the two profiling approaches to the respective other datasets. As we have GOESA and ACALOS available to characterize speech intelligibility and loudness perception, it would be interesting if the profiling approach of (18) also estimates speech intelligibility and loudness as the two distortion dimensions based on our data. Vice versa, the application of our approach to the BEAR test battery would generate a certain number of profiles, which could be compared to the profiles obtained in this study (and thereby to a comparison and potential combination of datasets), as well as reveal measurement combinations leading to sub-classes of the four auditory profiles of (18).

### Limitations of the profiling approach

Despite the advantages of our purely data-driven profiling approach, certain limitations persist. At the current stage, the profiling approach can detect plausible patient subgroups in data sets. This property generalizes also to further data sets containing different sets of measures and a different patient population. A restriction in the application of the current profiling pipeline to additional databases is the current requirement for continuous or at least ordinal features. Relevant audiological measures may, however, also be categorical with no inherent ordering. Thus, to also incorporate these measures, the current pipeline would need to be adjusted to also allow for categorical features.

The ability to detect differences in patient groups also depends on the sample size, the contained measures, as well as the presence of distinctive patient groups within the data set. If sample sizes are small, a smaller number of patient groups may be detected in the data sets, which in turn, would be defined by broader ranges across measures. At the same time, this could result in an increase in profiles, each containing only a few patients. This, however, would indicate that the underlying data set is not suitable for the herein proposed profiling approach, as nearly no similarities between patients could be detected. In such a case, it would not be certain whether a profile corresponds to a patient group that could also be identified in larger datasets, or whether it corresponds to outliers in the analyzed data set. Likewise, if only a few measures are contained in new data sets, not all existent distinctions between patients may be detected. Instead, only distinctions regarding the included measures would be available. Combining profiles generated on further data sets with the current profiles may, thus, prove difficult. An estimate of profile “conciseness” could tackle this challenge. This estimate could refer to the average similarity of patients within a profile regarding relevant measures. The similarity between patients with broader profiles will be smaller than the similarity between profiles with smaller ranges across audiological measures. As a result, the conciseness estimate could indicate if the generated profiles on the new data set only result in a coarse grouping of patients. It could then be analyzed, whether the coarse grouping could be explained by a mixture of already available auditory profiles. This would, however, require an overlap between audiological measures across the profiles. If the profiling pipeline is applied to a data set with low overlap regarding measures, the generated profiles would have to be interpreted separately from the current set of profiles, until a relation between measures has been established. This could either occur *via* available knowledge or by analyzing a data set that contains an overlap between the measures of interest. Regardless, newly generated profiles on further data sets would first need to be analyzed in terms of general audiological plausibility.

At the same time, the relevance of the distinctions between patient groups, in general, and for clinical practice needs further evaluation. This could either comprise asking experts to rate the plausibility and clinical applicability of the distinctions between the profiles or incorporating expert knowledge from other approaches toward patient characterization. The Common Audiological Functional Parameters (CAFPAs) by Buhl et al. (21), for instance, provide an expert-based concept of describing patient characteristics; and in Saak et al. (20), regression models were built to predict CAFPAs based on features that are also available for the current auditory profiles. Hence, the predicted CAFPAs would be available as additional descriptive information for the profiles generated in this study, and a consistency check to previous CAFPA classification (60) could be obtained by analyzing the same data set from different perspectives (i.e., analysis tools). In that way, both approaches provide complementary insights, and both contribute to future combined analysis of different audiological databases. As a result, physicians' trust toward applications (e.g., clinical decision-support systems) using the auditory profiles could be enhanced, which has shown to be a relevant factor in the adoption of such systems in clinical routine (61). Additionally, it can be assumed that the inclusion of more severe patient cases, e.g., with indications for a cochlear implant, could enhance the current profiles toward more extreme profile representations. Currently, profiles can be mostly assigned to mild to moderate hearing loss. With the inclusion of further data sets, containing a higher prevalence of severe patient cases, this aspect could be addressed.

## Application and outlook

The herein proposed profiling approach serves as a starting point for uncovering patient groups and patient presentations across audiological measures for the increasingly available amount of larger data sets. Consequently, the proposed profiling approach needs to be applied to additional data sets, which include more severe and diverse patient populations, as well as additional audiological measures to cover further important factors of hearing loss (e.g., binaural and central components). The set of auditory profiles would need to be updated after the inclusion of every further data set by either merging similar generated profiles or adding new profiles. In that way, it would conclude in a final set of auditory profiles, if generated profiles converge. This means that generated profiles on new datasets are already contained in the set of defined auditory profiles and no new information is added, thus, resulting in a final set of auditory profiles describing the audiological patient population.

If the generated auditory profiles describe the audiological patient population, they could be used in a variety of applications due to their flexibility. The profiles could efficiently summarize patient information for a clinical decision-support system. Likewise, they could also support mobile assessments of patients, in e.g., a “virtual hearing clinic.” If patients are tested on the measures used for the classification models (or appropriate mobile implementations of those measures, ensuring that measurements near the hearing threshold are feasible in realistic environments) they could be classified into a profile. In a clinical decision-support system, physicians could then be provided with statistical insights into patients' hearing statuses, whereas in virtual hearing clinic patients themselves could receive information regarding their hearing statuses. To also provide diagnostic decision-support as well as aided benefit predictions, however, data from additional data sets containing these measures need to be incorporated into the current profiles. A metric allowing for the combination or separation of profiles, if new profiles are generated on additional data sets, hence, needs to be defined.

After the final set of auditory profiles has been defined, it would also be of interest to define a minimum set of tests that allow for adequate classification of patients into the profiles across data sets. This could highlight the audiological measures that are most relevant across all profiles. Likewise, the profiles could contribute to the selection of the next to-be-performed measures for characterizing the patients. If classification models are available for all measurement combinations, measures leading to the best discriminatory performance across profiles could be selected next. This, in turn, could reduce the testing time of the patients, as well as support the derivation of test batteries covering all relevant aspects of hearing deficits, as in (5, 6), by highlighting the most important measures.

## Conclusion

The proposed data-driven profiling approach resulted in 13 distinct and plausible auditory profiles and allows for efficiently characterizing patients based on the interrelations of audiological measures. All patients are characterized and patient groups with certain characteristics, such as asymmetry, are not excluded. Due to the profiles' flexibility by being defined on the contained patients' measurement ranges, profiles could be added or refined, given insights derived from applying the profiling approach to additional data sets. The profiles concur with other profiling approaches but are able to detect differences in patient groups regarding measurement ranges in more detail than hitherto available approaches.

New patients can be adequately classified into the auditory profiles for 12 of the 13 auditory profiles. For 10 profiles, both high precision and sensitivity were achieved (>0.75), and for two profiles, low to medium sensitivity and high precision were achieved, and for one profile no classification could be achieved due to the profiles' small sample size. Since the classification model was based on a reduced set of measures often available in clinical practice in Germany (GOESA, ACALOS, air-conduction audiogram, and age), clinicians could use the auditory profiles even without performing a complete audiological test battery, if a quick classification with less clinical detail is required. Likewise, all measures required for classifying patients into the auditory profiles are potentially available also on mobile devices, facilitating mobile assessments of the patient.

The proposed profiling approach depends on the underlying data set in terms of the number of profiles or the covered range of patients. Its properties such as flexibility, not being tailored toward a specific outcome, or ability to handle incomplete patient data, however, generalize to other data sets including additional measures. Appropriate means to combine profiles generated across data sets need to be defined.

Future research should extend the profiling toward integrating different data sets with more severe and diverse patient cases. In addition, binaural measures should be included, as well as aided data to investigate hearing device benefits with the profiles.

## Data availability statement

The data analyzed in this study was obtained from Hörzentrum Oldenburg gGmbH, the following licenses/restrictions apply: According to the Data Usage Agreement of the authors, the datasets analyzed in this study can only be shared upon motivated request. Requests to access these datasets should be directed to MB, mareike.buhl@uni-oldenburg.de and SS, samira.saak@uni-oldenburg.de. The analyses scripts can be found here: Zenodo, https://zenodo.org/, https://doi.org/10.5281/zenodo.6604135.

## Author contributions

SS conducted the data analysis which was continuously discussed with all authors and drafted the manuscript. All authors conceptualized, designed the study, and contributed to the editing of the manuscript.

## Funding

This work was funded by the Deutsche Forschungsgemeinschaft (DFG, German Research Foundation) under Germanys Excellence Strategy −EXC 2177/1 – Project ID 390895286.

## Conflict of interest

The authors declare that the research was conducted in the absence of any commercial or financial relationships that could be construed as a potential conflict of interest.

## Publisher's note

All claims expressed in this article are solely those of the authors and do not necessarily represent those of their affiliated organizations, or those of the publisher, the editors and the reviewers. Any product that may be evaluated in this article, or claim that may be made by its manufacturer, is not guaranteed or endorsed by the publisher.
